# Soil contamination in Europe unveiled: A review of pesticides and metabolites to watch

**DOI:** 10.12688/openreseurope.20475.1

**Published:** 2025-08-27

**Authors:** Raquel Carvalho, Paula Guedes, Eduardo P. Mateus, Vera Silva, Pavlos Tyrologou, Nikolaos Koukouzas, Alexandra B. Ribeiro, Nazaré Couto

**Affiliations:** 1 CENSE – Center for Environmental and Sustainability Research & CHANGE - Global Change and Sustainability Institute, NOVA School of Science and Technology, NOVA University Lisbon, Campus de Caparica, 2829-516 Caparica, Portugal; 2 Department of Environmental Science, Aarhus University, Frederiksborgvej 399, 4000 Roskilde, Denmark; 3 Soil Physics and Land Management Group, Wageningen University, 670PB Wageningen, The Netherlands; 4 Chemical Process & Energy Resources Institute CPERI, Centre for Research & Technology Hellas CERTH, Maroussi, 15125, Athens, Greece

**Keywords:** soil contamination, pesticide residues and metabolites, soil health

## Abstract

Soil is multifunctional and fundamental for both humans and ecosystem health. However, it faces growing threats from contamination, particularly from pesticides. In this review, pesticide contamination trends across Europe were assessed by analysing published data from 5193 sampled soils collected between 2015 and 2022. By raking pesticides based on detection frequency, persistence and toxicity, key concerns were brought to attention, including the presence of banned substances, such as p,p’-DDT (detected in 31% of sampled soils) and Atrazine (17%), as well as high detection rates of currently approved pesticides like Boscalid (36%) and Epoxiconazole (32%).

Results also revealed regional contamination patterns and differences. Greece and Poland presented a strong association with non-approved pesticides. The presence of these substances, although long banned, raises concerns about their origin, persistence and potential cross-border pollution. In contrast, Portugal appears to be more associated with currently approved pesticides. Furthermore, metabolites like AMPA, a degradation product of Glyphosate, was detected in 44% of soils, which highlights the contribution of metabolites in long-term contamination risks. The metabolite 1,2,4-triazole has been proposed as a potential indicator of soil pesticide contamination, which could enhance monitoring and reduce associated costs.

These results point out the limitations of currently regulatory frameworks, which often fail to account for environmental transport, persistent residues, and policies related to pesticide distribution across countries. To protect soil health, monitoring programs and remediation strategies are necessary. Establishing more comprehensive legislation for both active substances and their breakdown products is essential to mitigate long-term contamination risks.

## Plain language summary

Healthy soils are vital for our everyday lives - they are essential for growing the food we eat, to protect our environment, and to support the life on Earth (in alignment with “SDG 2 - Zero Hunger”, “SDG 12 - Responsible Consumption and Production” and “SDG 15 - Life on Land” respectively).

This work analysed the data from over 5,000 soil samples collected across Europe between 2015 and 2022 to perceive where and how pesticide pollution affects European territory. The results give cause for concern. Many of the soil samples had the presence of pesticides, including some that have been banned for years, like DDT and atrazine, as well as others that are still allowed, like boscalid and epoxiconazole. Specific regional contamination patterns were also found.

Some countries, like Greece and Poland, were found to have a strong association with unapproved pesticides, raising concerns about their use or persistence. The study also found “leftover chemicals” from pesticides, specifically breakdown products. These are what’s left after the pesticide starts to degrade – and they can be just as harmful as the original pesticides. One such chemical, 1,2,4-triazole, may be useful as an indicator and serve as a warning sign of soil contamination. The findings show that pesticides can move between countries through trade or the environment and persist in the soil for a long time.

The findings highlight the need for better and more comprehensive soil monitoring, as well as frameworks that better protect the health of soil, humans and ecosystems.

## Introduction to the context

### Soil

Soil is an extremely thin layer, representing only one ten-millionth of the Earth's entire radius ^1^. Yet it plays a vital role in our lives. Soil is ‘multifunctional’, performing multiple key functions that both humans and the planet depend on for survival.

These functions include biomass production, in which the soil acts as a medium for plant and produce development. It plays a crucial role in carbon storage, helping to mitigate climate change. It recycles nutrients like nitrogen, phosphorus, and potassium, making them available for plant uptake. Soil also impacts water regulation involving not only water storage but also purification, by removing contaminants. Soil provides habitat for diverse organisms, including bacteria, fungi, and invertebrates, contributing to biodiversity ^2, 3^. There are estimates suggesting that a single handful contains 10 to 100 million of them ^4^. These life forms are crucial to soil functions. Organisms like earthworms create tunnels, increasing soil porosity and consequently aeration and water infiltration. This helps decrease soil erosion by 50%. Insects like beetles and ants are able to transport nutrients. Invertebrates like earthworms and millipedes are capable of transforming decaying material and minerals into usable forms, increasing soil fertility. Along with this, many of these organisms are capable of controlling pests and diseases ^5^.

Soil health is defined as the continued capacity of soils to deliver these multiple essential functions ^3^. Healthy soils are essential for achieving the United Nations Sustainable Development Goals (SDGs) by supporting food supply and security (SDG 2), clean water (SDG 6), biodiversity and ecosystem services (SDG 15), and climate action through carbon sequestration (SDG 13). Despite its importance, soil health is threatened by, among others, improper use and management, leading to soil degradation ^6^. It is estimated that 33% of the world's soil is already degraded and that 60–70% of the soils of Europe are in an unhealthy state ^7, 8^. Furthermore, UNESCO (United Nations Educational, Scientific and Cultural Organization) estimates that this number will reach 90% by 2050 ^9^.

In Europe, soil degradation has distinct characteristics. While much of the continent struggles with erosion and contamination, industrialized and densely populated regions also face soil sealing due to urbanization and expanding infrastructure. In the Mediterranean, erosion is the primary form of degradation, while central, western, and northern Europe are faced with significant soil contamination, with an estimated of 2.5 million contaminated sites ^10, 11^. Thus, soil contamination poses a major threat, undermining SDGs goals. Contaminants disrupt ecosystems, harm human health (SDG 3), and degrade land ^12^.

These contaminants can originate from various sources, including agricultural activities, industrial processes, urban waste, mining, and effluents, and include, but are not limited to, heavy metals, volatile organic compounds (VOCs), polycyclic aromatic hydrocarbons (PAHs), personal care products, pharmaceuticals, and pesticides ^13– 17^.

The increased demand for food production has resulted in the intensification of agricultural practices, which has destroyed habitat and contaminated soil through pesticide use. Soil contamination by agrochemicals, particularly pesticides, is a growing concern due to its harmful effects. While pesticides play an important role in modern agriculture, their overuse or improper application can disturb soil ecosystems. These effects are usually shown in key soil health indicators, including reductions in organic matter content, soil porosity, microbial biomass, and biodiversity ^1^.

### Objective of this review

Pesticides endanger us all, making it crucial to address, prevent and remediate soil contamination. Monitoring plays a critical role, since it allows for the detection of contamination levels, tracks the efficacy of treatment strategies, and helps ensure that residual pesticides do not reaccumulate over time. Without regular monitoring, the success of remediation efforts cannot be fully assessed, and the possibility for recurring contamination remains high.

In addition, monitoring pesticide contamination in soil is crucial for achieving several SDGs ^12^. It directly supports SDG 2: Zero Hunger by ensuring soil remains healthy and able to produce food. It also aligns with SDG 3: Good Health and Well-being, since contaminated soil might introduce harmful chemicals into food and water sources. It contributes to SDG 6: Clean Water and Sanitation by preventing pesticide runoff into water bodies. Responsible pesticide use promotes SDG 12: Responsible Consumption and Production. It also supports SDG 13: Climate Action, as healthy soils play a critical role in carbon sequestration and climate change. Lastly, it helps achieve SDG 15: Life on Land by preserving biodiversity.

This review aims to investigate pesticide contamination of European soils and to identify which are the most frequently detected ones across this continent. Understanding the prevalence and possible trends of these contaminants is crucial for informed decision-making, helping to prioritize efforts where they are needed most. By offering a comprehensive overview, this paper aims to guide future research on pesticide monitoring and remediation, ultimately preserving soil health.

## Introduction to pesticides

### Pesticides

Pesticides are substances, or mixtures of substances primarily used in agriculture to prevent and destroy pests that endanger crops. These chemical or biological substances are the foundation of modern agriculture, helping to meet the demands of a growing population. The EU Pesticides Database lists more than 1400 active substances, of which 433 are currently approved for use ^18^. These figures demonstrate our dependence on such products. Although pesticides have the advantage of controlling pests, not all but most, also pose the disadvantage of being harmful not only to their intended targets but also to humans and other living beings ^19^.

Considering potential impacts on humans, pesticides can enter the human body through the skin, eyes, mouth, and respiratory system. While acute poisoning is rare, chronic toxicity from continuous exposure to low quantities of pesticides is common ^19^. Pesticides are linked to various health issues, including asthma, dermatitis, reproductive dysfunctions, neurobehavioral disorders, and even cancer ^19^. Pesticide residues have also been detected in human breast milk raising concerns about the health effects in children ^20^. According to WHO (World Health Organization), there are five classes of toxicity: extremely hazardous (Ia), highly hazardous (Ib), moderately hazardous (II), slightly hazardous (III) and unlikely to present acute hazard (U) ^21^.

Pesticides are categorized into several types: insecticides, which target insect pests; fungicides, which prevent and control fungal infections in plants; and herbicides, which eliminate unwanted vegetation, such as weeds. They can also be classified according to their toxicity and chemical structure. Some are inorganic, but most are organic. The main classes commonly reported are organochlorines, organophosphates, carbamates, and pyrethroids ^22^.

Pesticides are usually sold as a mixture of an active substance and inert ingredients. Inert ingredients are added to improve stability and achieve effective, safe and economic use of pesticide formulations ^23^. These are called ‘inert’ because they are not meant to have a direct effect on pest control. However, just like the active ingredients, although the inert ingredients provide certain advantages, they come with risks. Studies have demonstrated that commercial pesticide formulations are more toxic than their pure active ingredients ^24^. Additionally, so-called inert ingredients have been found to enhance the absorption of the active ingredient—not only by the target organism but also through the skin of individuals who come into contact with them ^25^.

### Pesticides in the environment

**Figure 1.**
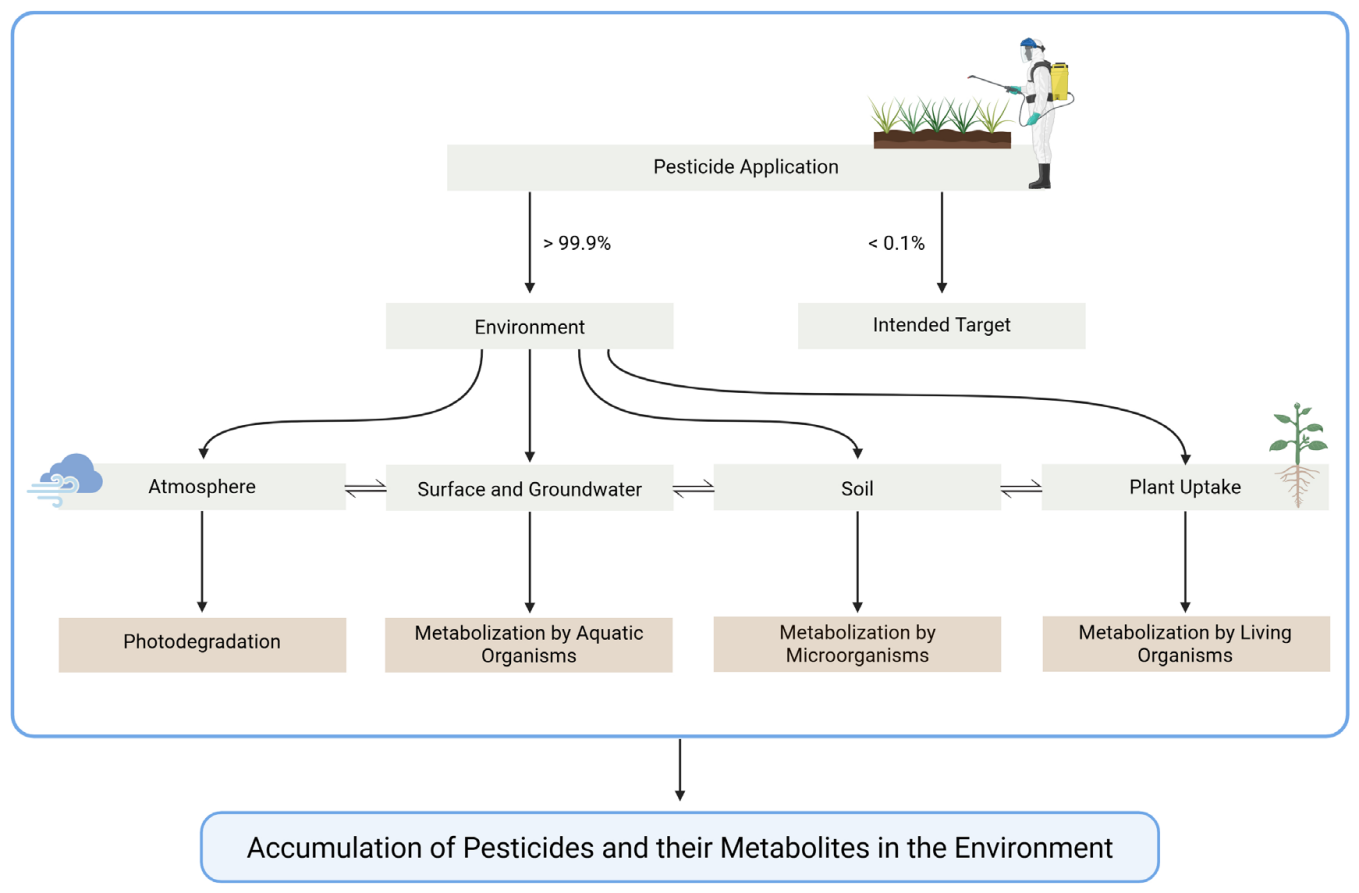
Simplified representation of the main distribution pathways of pesticides. Created in BioRender. Carvalho, R. (2025) https://BioRender.com/ita1d60.

It is estimated that under worst-case scenarios, less than 0.1% of applied pesticides reach their intended target ^26^. The remainder can degrade or disperse, potentially contaminating other areas, including the atmosphere and waterbodies. Pesticides can volatilize into the air, run off into surface water, leach into groundwater, be taken up by plants or soil organisms, or remain in the soil, representing the main pathways across environmental compartments ( Figure 1) ^27^.

During application, pesticides can deposit onto non-target and/or be immediately lost in the atmosphere. They can also volatilize post-application ^28^. Volatilization is influenced by soil properties (water content, organic matter), pesticide properties (vapour pressure, solubility), and environmental factors (airflow, temperature) ^29^. The main issue is the distribution of pesticides across the planet. Pesticides can travel long distances, though dilution and degradation reduce their risk. However, some may deposit, posing exposure risks to humans and animals ^21, 22^.

Pesticides enter water sources mostly via runoff and leaching. Runoff occurs when water flows across the soil, carrying pesticides into rivers, lakes, and seas. Leaching happens when pesticides move through permeable soils, reaching groundwater ^30, 31^.

This highlights how pesticide use contaminates and disrupts ecosystems, a problem exacerbated by the accumulation of both pesticides and their metabolites in the soil ^19^.

### Metabolites

Degradation is the process through which a pesticide is broken down into smaller molecules. This process can occur through 2 pathways: chemical and microbiological. Reactions such as photolysis, hydrolysis, oxidation and reduction are examples of chemical degradation. Biological degradation takes place when soil microorganisms are involved in the pesticide's metabolism ^32^.

Biological metabolism typically follows a three-phase process. In Phase I, the parent compound undergoes oxidation, reduction, or hydrolysis, to form a product that is generally more water-soluble and usually less toxic. Phase II involves the conjugation of the pesticide or its metabolite with molecules such as sugars, amino acids, or glutathione, further enhancing water solubility and reducing toxicity ^33^. Phase III involves the excretion of these metabolites, or in case of plants, the transformation of these into secondary conjugates that are non-toxic ^33, 34^.

The rate of degradation can be characterized by the half-life (DT50), which represents the time required for the concentration of a compound to be reduced by half through degradation ^35^. DT50 depends on the properties of the pesticide, characteristics of the soil, climatic conditions, and the number and type of microorganisms present ^36^. Pesticides with DT50 shorter than 30 days are classified as non-persistent, such as captan (2–8 days) and malathion (1 day). Those with a half-life between 30 and 100 days are classified as moderately persistent, like benomyl (67 days) and diuron (90 days). When this time is greater than 100 days, they are considered persistent, such as terbacil (204–252 days) and lindane (120 days) ^36, 37^.

When half-lives are assessed under controlled laboratory conditions, they often give rise to higher values compared to those observed in the field. This occurs because, under field conditions, multiple degradation pathways are at play. Also, losses by volatilisation, runoff and leaching are happening. These factors, while not degradation pathways *per se*, significantly contribute to the loss of compounds in the soil ^27^.

**Figure 2.**
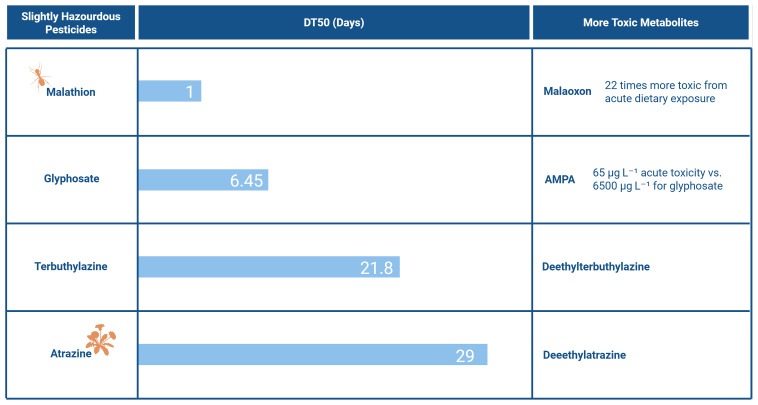
Example of pesticides and their respective DT5O, Toxicity (WHO Classification) and metabolites with higher toxicity ^32, 38, 39^. Created in BioRender. Carvalho, R. (2025) https://BioRender.com/d9iwzn2.

Although, during metabolism, pesticides are usually transformed into less toxic compounds than the parent compound, it does not mean that the opposite cannot happen. In some cases, the metabolites are more toxic ( Figure 2). Even when metabolites are less toxic than their parent compound, they can still harm the environment, for instance, by exhibiting higher mobility in soil. This issue is further exacerbated by the fact that multiple metabolites can be present in the soil and may be present in substantial quantities ^32^.

The formation of diverse metabolites during pesticide degradation can vary based on the degradation pathway and the type of organism involved, resulting in a complex mixture of substances ^36^. For instance, trifluralin alone can yield at least 28 different metabolites ^40^.

### Pesticide legislation

Pesticide use is regulated across the world ^41^. Within the European Union (EU), Regulation (EC) No 1107/2009 governs the placing of plant protection products on the market. This regulation establishes the criteria that plant protection products must meet to be approved in the EU, including toxicological effects, environmental impact, persistence, and bioaccumulation, ensuring protection for human and animal health and the environment ^42, 43^. While this regulation provides a framework for pesticide approval and use, it is complemented by Directive 2009/128/EC, which establishes a framework for the sustainable use of pesticides ^43^. This directive promotes integrated pest management (IPM), restricts aerial spraying, mandates training and certification for pesticide users, and sets rules to protect water and sensitive areas. Unlike Regulation (EC) No 1107/2009, which applies directly across all Member States, Directive 2009/128/EC requires national implementation, allowing countries to determine how best to achieve its objectives.

Although these regulations and directives focus on safeguarding human and environmental health, they do not fully address the complexities posed by pesticides, particularly their persistence and behaviour in different environmental compartments. EU-wide, it is not required for pesticide residues to be monitored in soil, but such monitoring is mandatory for water under the Water Framework Directive (2000/60/EC) ^44^. Regarding drinking water, the Drinking Water Directive (98/83/EC) imposes a maximum allowable concentration of 0.1 μg/L for any individual pesticide and 0.5 μg/L for total pesticide concentration ^45^.

Even though soil monitoring is not mandatory, a study based on data from LUCAS 2015 (Land Use/Cover Area Frame Survey), which analysed 317 agricultural topsoil samples across Europe, found that 83% of the samples contained at least one pesticide residue ^17^. A more recent study, based on LUCAS 2018, which analysed over 3,000 sampled soils, determined that 74.5% of sites had at least one pesticide residue and 57.1% contained mixtures of two or more substances ^46^. The planned European Soil Health Law may tackle this issue by defining scientific and legal criteria for healthy soil, establishing obligations for soil monitoring at the EU level, and ensuring harmonized assessment methods across Member States ^47^. This law is expected to improve soil protection policies by addressing contamination, biodiversity loss, and soil degradation. Additionally, the EU has set ambitious goals under the Farm to Fork Strategy to reduce the use and risk of chemical pesticides by 50% by 2030 ^45^.

Despite the implementation of Regulation (EC) No 1107/2009, some non-approved pesticides are exported to non-EU countries, increasing the risk that imported food consumed within the EU may still contain these residues ^48^. The European Food Safety Authority (EFSA) monitors compliance with maximum residue levels (MRLs) set under Regulation (EC) No 396/2005—limits established to protect consumers based on good agricultural practice (GAP) ^49^. However, this does not eliminate the possibility of contamination at low levels deemed non-harmful. Additionally, Member States can issue emergency authorizations for active substances that are not approved at the EU level, potentially contributing to further contamination and posing long-term environmental risks ^48^.

As mentioned, pesticides are not applied individually but are used in complex mixtures of active substances and inert ingredients. Therefore, legislation should not focus solely on active substances. However, in the EU, toxicity testing is conducted on a single commercial formulation per active ingredient ^50^. This poses a challenge, as there can be hundreds of different formulations for one active ingredient, potentially leading to an underestimation of toxicity ^51^. Furthermore, Regulation (EC) No 1907/2006 requires only co-formulants with specific human hazard statements to be reported, while others remain undisclosed as proprietary information ^50, 52^. This lack of transparency makes it difficult to fully assess how inert ingredients interact with active substances, affecting both dispersion and environmental impact ^50^.

## Material and methods

### Literature review

A systematic literature review was conducted, examining articles on soil organic contaminants, focusing on the ones reporting pesticides and the degradation products.

**Figure 3.**
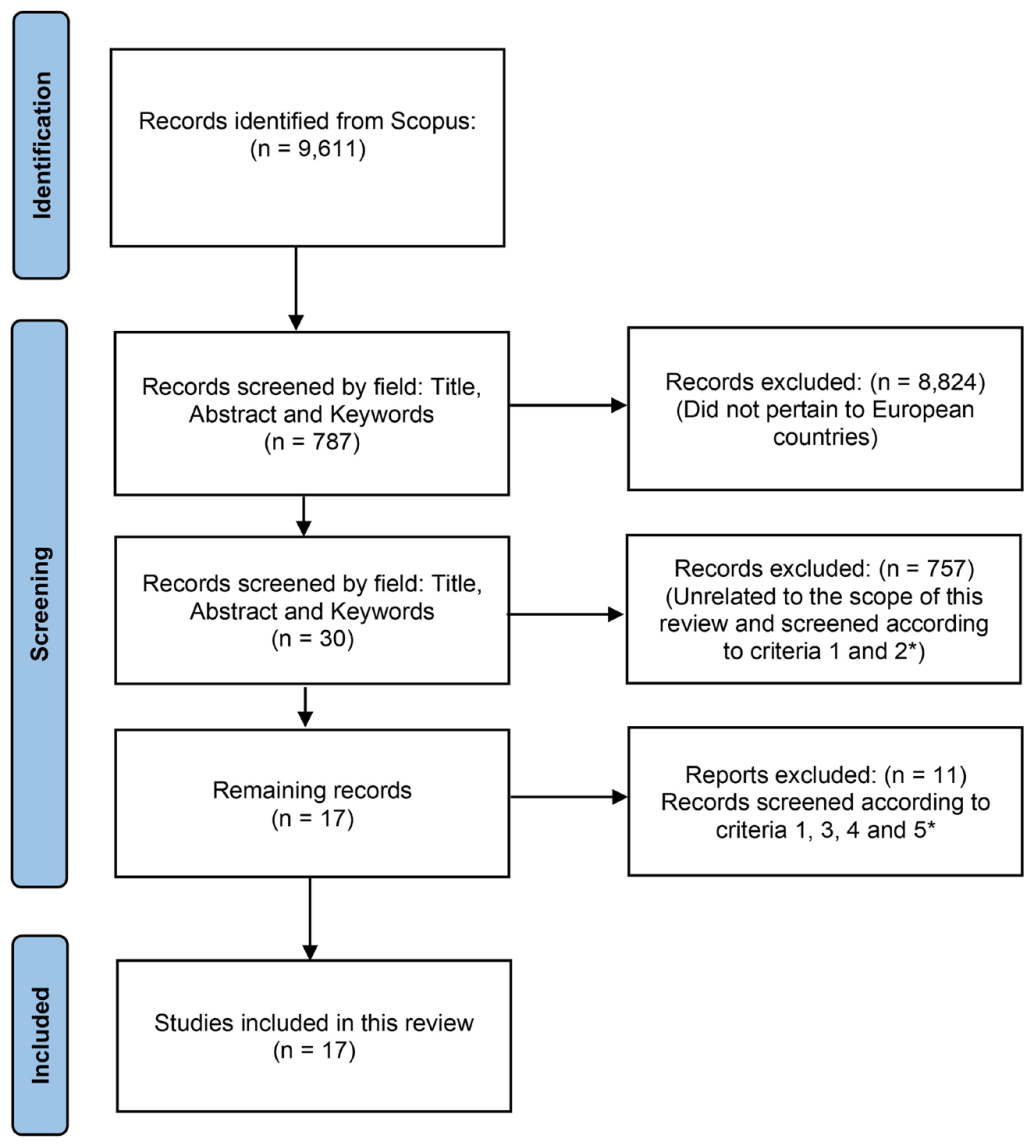
Diagram of the methodology used to include/exclude records to be used in this review. * Criterion 1: Records must study multiple pesticides rather than focusing on only one or two and their metabolites. Criterion 2: Records must be written in English. Criterion 3: The study must indicate the soil characteristics (e.g., pH, % organic matter), the land use (such as the specific crop), and the country where the study was conducted. Criterion 4: The study must specify the frequency (i.e., number of sampled soils) in which each pesticide was found, whether directly or indirectly. Criterion 5: The analytical method must be detailed, including sample collection procedures, time of collection, sample preparation, and chromatographic conditions.

The dataset was extracted from the Scopus literature database assessed in June 2024, covering publications from 2019 to 2024. Only these five years were considered to ensure the most recent data on contamination. The search was conducted using the English keywords “soil” and “pesticides”. The records were screened by title, abstract, highlights, and keywords to exclude any that did not pertain to European countries, ensuring our review focused solely on EU relevant data. The records were also screened to remove any that were not within the scope of this review ( Figure 3). They were also checked for five criteria. Criterion 1: Records must study multiple pesticides rather than focusing on only one or two and their metabolites. Criterion 2: Records must be written in English. Criterion 3: The study must indicate the soil characteristics (e.g., pH, % organic matter), the land use (such as the specific crop), and the country where the study was conducted. Criterion 4: The study must specify the frequency (i.e., number of sampled soils) in which each pesticide was found, whether directly or indirectly. Criterion 5: The analytical method must be detailed, including sample collection procedures, time of collection, sample preparation, and chromatographic conditions.

### Building lists of pesticides of concern

The 17 articles were used to create pesticide lists, which highlight the most frequently reported pesticides across Europe ^17, 53– 68^. Not only that, but it was taking into consideration their properties such as persistence (DT50) and toxicity, pinpointing which are the pesticides of concern to watch. A spreadsheet was created using various data sets to generate a final table. This table was based on the results from all seventeen studies, taking into account the number of sampled soils in which a particular active substance was detected and quantified, as well as the overall number of sampled soils analysed for that pesticide. A rule was established that only pesticides quantified in the studies were included, as some articles reported only the limit of quantification (LOQ) rather than the limit of detection (LOD).

To refine the selection, priority was given to the most frequently detected pesticides. In cases where multiple pesticides had the same frequency and were found in the same number of soils, or if one or both of these variables differed slightly, they were further evaluated based on their characteristics. Specifically, pesticides that were more toxic, more persistent in the environment, and had higher bioaccumulation potential were given priority. This approach ensured that the lists focused on substances posing the greatest potential risk.

**Figure 4.**
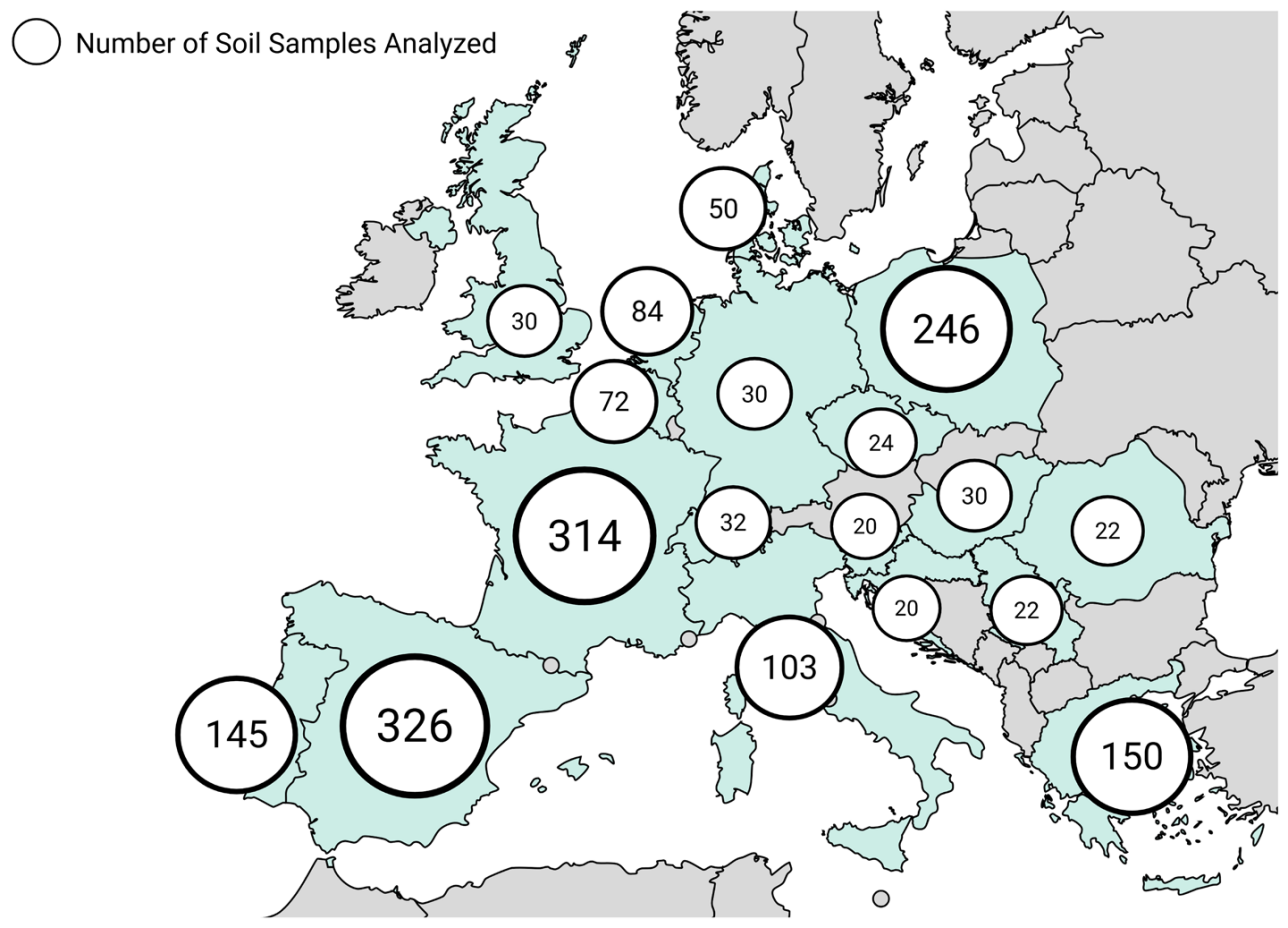
Spatial distribution of sampled soils reviewed ^17, 46, 53– 69^. Created in BioRender. Carvalho, R. (2025) https://BioRender.com/fc9pzv5.

The bibliographic analysis yielded information on the presence of pesticides in 1720 soils, across 18 countries, which was cross-referenced with data from the 2018 LUCAS covering 3473 locations, totalling 5193 soils analysed collected between 2015 and 2022 ( Figure 4) ^17, 46, 53– 68^.

### Data analysis: Principal component analysis

Principal component analysis (PCA) was used to analyse and visualize trends across countries based on reported pesticides. PCA was carried first with the pesticides that were reported across all selected countries and secondly with the non-approved pesticides only. The analysis was based on the frequency of pesticide occurrence. The two resulting data matrices were imported into the Unscrambler ^®^X version 10.5 software (CAMO Software, Oslo, Norway) for analysis. Scores plots were used to visualise country groupings, while loadings plots give insight into the pesticides that most influenced these patterns.

## Results and discussion

### Pesticides use across Europe

***Pesticides sales***

**Figure 5.**
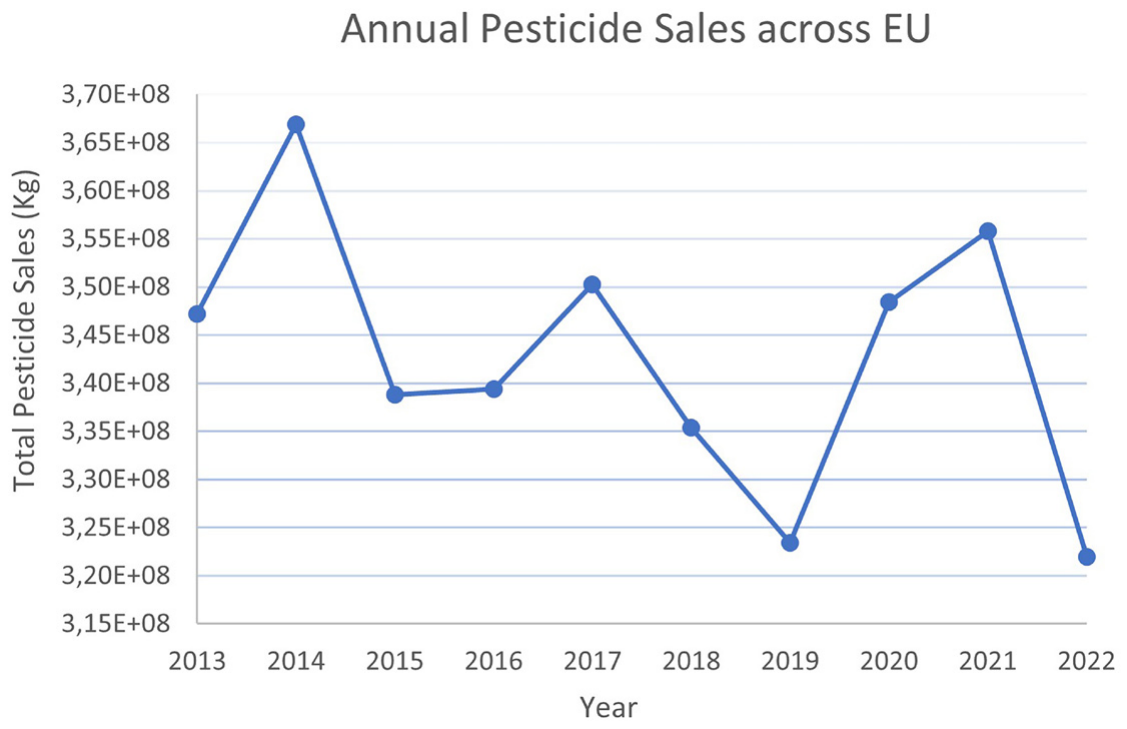
Annual pesticide sales across EU ^70^.

Across the EU, the sales of pesticides from 2013 to 2022 have oscillated starting at 347 kt in 2013 and peaking at 367 kt in 2014 ( Figure 5) ^70^. The lowest sales during this period occurred in 2022 with 322 kt, largely due to a surge in prices ^71^. A similar downturn was recorded in 2019, when sales dropped to 323 kt, a decrease attributed to adverse weather conditions, including drought ^45^.

During this ten-year period, France and Spain have consistently been the top two consumers of pesticides, with average annual sales of 69 kt and 73 kt, respectively. Fungicides and herbicides were the most sold categories, accounting for 42–49% and 32–36% of total pesticide sales, respectively ^70^.

**Figure 6.**
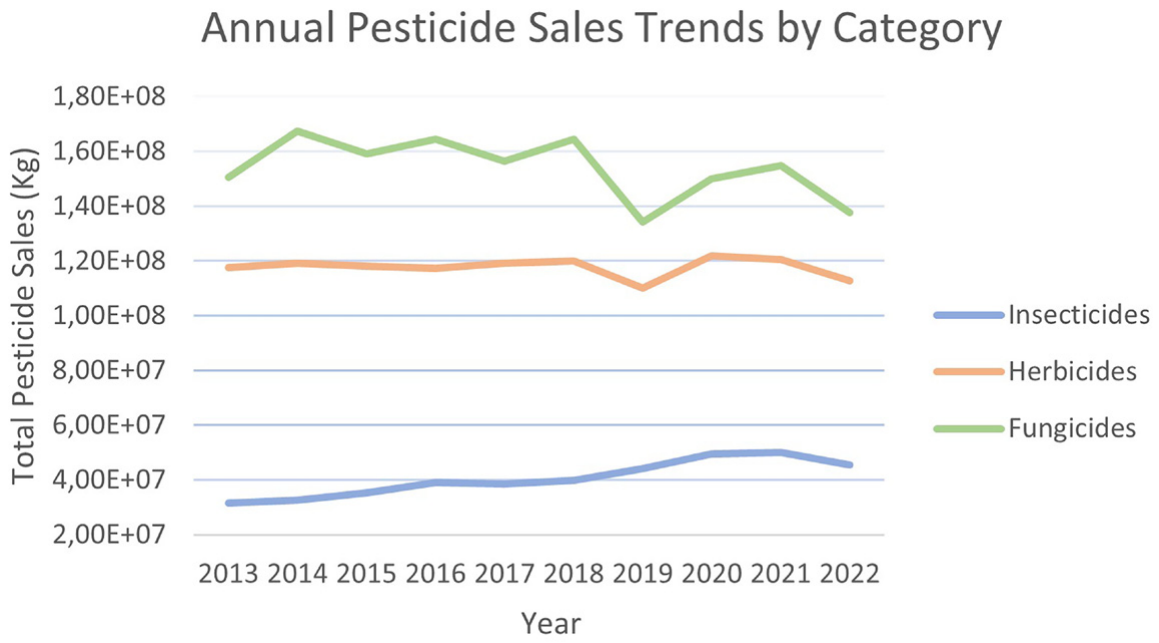
Annual pesticide sales trends by category ^70^.

A clear trend is visible in Figure 6: when total pesticide sales decreased ( Figure 5), fungicide sales generally followed the same pattern, while herbicide sales were less affected by these fluctuations. The same cannot be said for insecticides, as their sales have been steadily growing over the years. Insecticides have consistently been the last sold category, contributing only 9-14% of total pesticide sales, but have shown a notable increase, starting at 31 kt in 2013 and peaking at 50 kt in 2021 ^70^. Despite a decline in 2022, as seen across all pesticide categories, the overall growth in insecticide sales raises concerns about issues such as insecticide resistance ^72^.

***Pesticides of concern***

**Figure 7.**
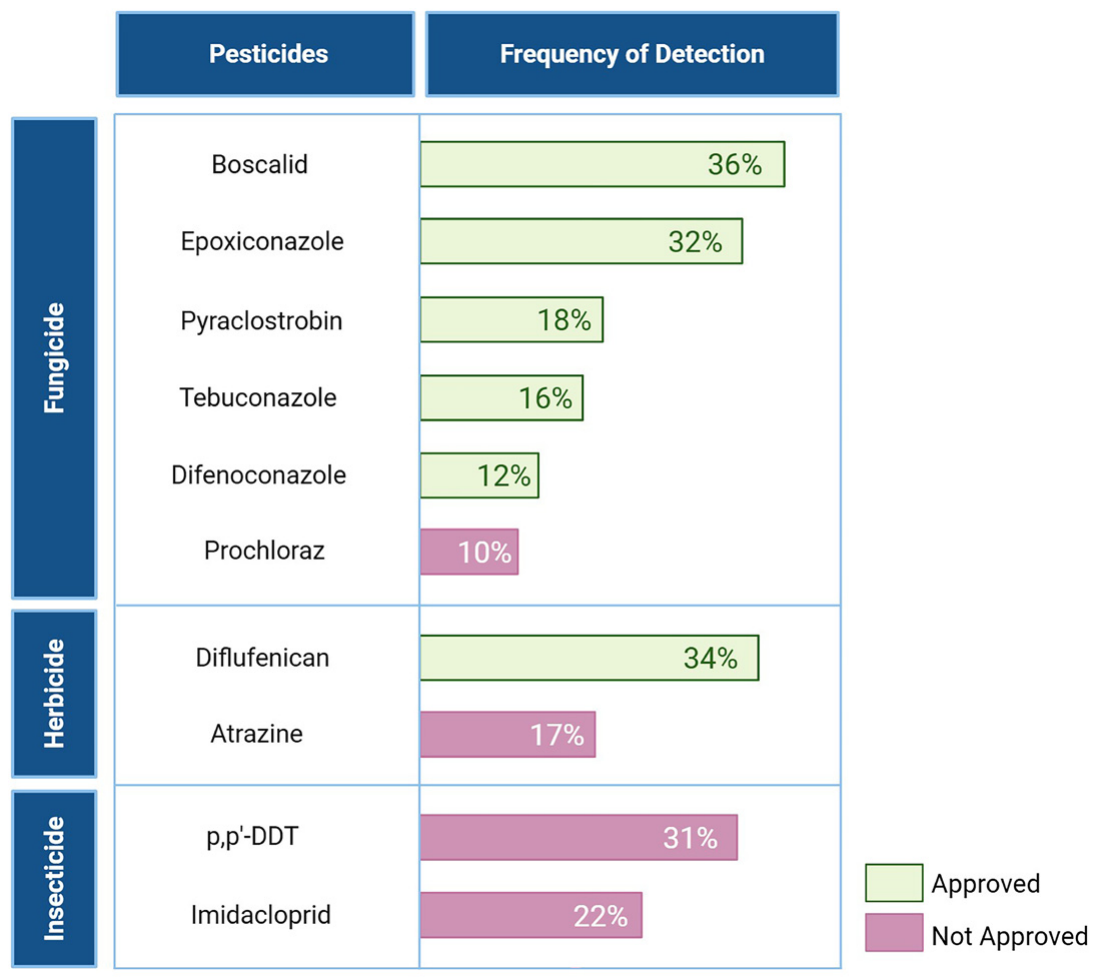
TOP 10 pesticides of concern across Europe, their characteristics and frequency detected ^17, 46, 53– 69^. Created in BioRender. Carvalho, R. (2025) https://BioRender.com/7d6kyus.

Given the overall sales trends, it is crucial to identify the specific pesticides driving these changes. Using data from seventeen studies and the 2018 LUCAS, pesticides were ranked based on their persistence, toxicity, and detection frequency, providing a clearer understanding of emerging substances of concern ^17, 46, 53– 68^. Sampled soils analysed in these studies were collected between 2015 and 2022, offering insight into recent pesticide contamination trends. A total of 455 pesticides were considered in the study ^17, 46, 53– 68^. However, many of these pesticides were analysed in only a single article, limiting the ability to assess their distribution across Europe due to a low number of sampled soils or regional focus. Only pesticides studied in multiple articles were included in the final analysis, ensuring a more comprehensive understanding of their presence and distribution. The pesticides listed in Figure 7 were referenced in multiple studies, confirming their widespread analysis and inclusion in the assessment ^17, 46, 53– 68^.

Starting with an analysis of pesticide presence across the EU, Figure 7 shows that most of the pesticides on the TOP 10 pesticides of concern across Europe list are fungicides, in line with sales trends. Notably, two insecticides — p,p’-dichloro-diphenyl-trichloroethane (p,p’-DDT) and Imidacloprid — and two herbicides — Atrazine and Diflufenican — also feature on this list. Additionally, the results from the 2018 LUCAS confirm that pesticide distribution in the sampled soils aligns with the distribution of annual sales ^46^.

While fungicides like Boscalid and Epoxiconazole (detected in 36% and 32% of soils, respectively) are still approved for use, older insecticides like p,p’-DDT, banned for many years, continue to persist in the environment, being detected in 31% of the sampled soils analysed. Prochloraz, Atrazine, and Imidacloprid, all non-approved pesticides, also feature in the top ten list. The inclusion of Prochloraz and Imidacloprid is not surprising, given their bans in 2021 and 2018, respectively, during the interval when the sampled soils were collected ^73, 74^.

The 2018 LUCAS highlights that the insecticide Imidacloprid, along with fungicides such as Epoxiconazole, Difenoconazole, and Boscalid, are key drivers of toxicity in soil. While Pyraclostrobin, Diflufenican, Atrazine, and p,p’-DDT — the other pesticides in the top 10 list of concern across Europe — were not identified as major contributors to soil toxicity in the survey, they were still detected in the sampled soils of this study ^46^.

These findings highlight the persistence of certain pesticides in soil despite bans and regulatory measures, demonstrating that past agricultural practices left a legacy and continue to affect soil quality. The 2018 LUCAS also concluded that regulations on pesticide sales have not prevented the presence of non-approved substances in EU soils, as multiple of these were detected ^46^. At the same time, currently approved substances like Boscalid and Diflufenican contribute to ongoing soil contamination, indicating that currently used pesticides have the potential to accumulate in the environment.

**Pesticides across countries**

**Figure 8.**
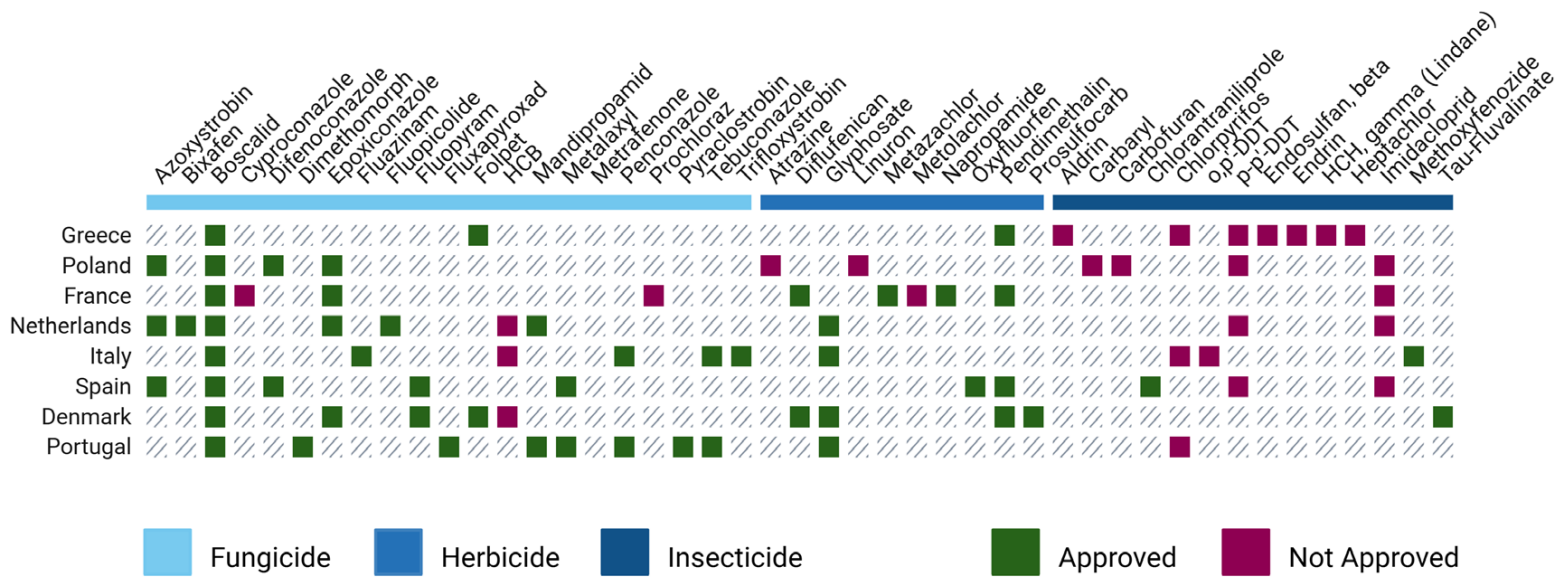
TOP pesticides of concern across different European countries and their regulatory status ^17, 46, 53– 69^.

**Figure 9.**
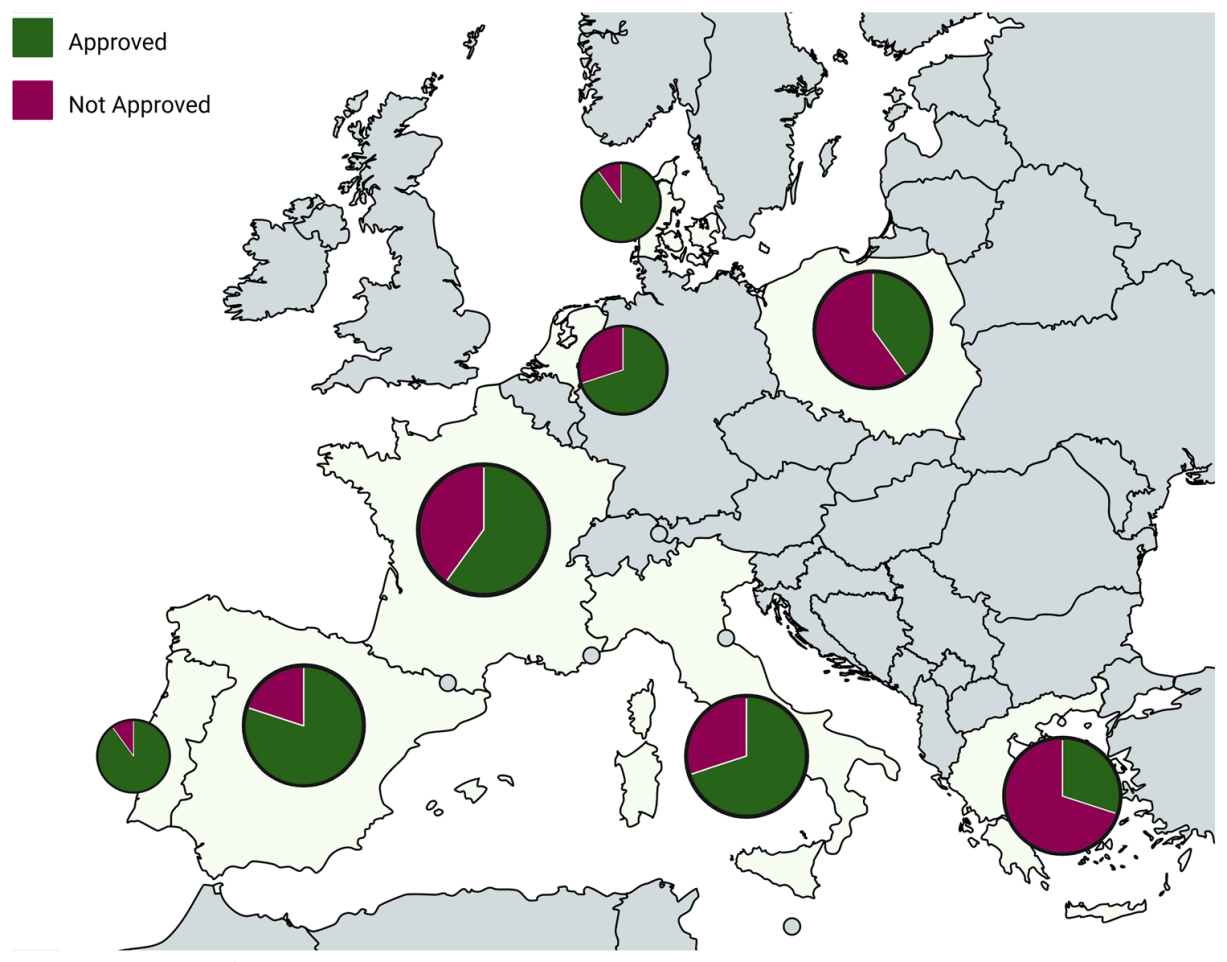
Spatial distribution of the ratio of approved vs. non-approved pesticides in the top 10 pesticides of concern across Europe ^17, 46, 53– 69^. Created in BioRender. Carvalho, R. (2025) https://BioRender.com/5epgy0s.

Having established a broad overview of pesticide trends throughout Europe, it is valuable to examine how these patterns play out on a national level. For countries where more than one study was conducted (i.e., multiple articles referenced and analysed for the same country), "Top 10 Pesticides of Concern" lists were also created ( Figure 8 and Figure 9; refer to extended data - Tables S1–S8).

Having in mind that overall, while country-level data offer valuable insights into regional differences, their lower sample sizes introduce limitations in making reliable conclusions ( Figure 4). Still, some notable patterns emerge. Among all countries, Greece stands out for its high presence of non-approved pesticides featuring in its list, like Aldrin (42%), Heptachlor (44%), and Endosulfan beta (20%). Despite existing differences between countries, there are several commonalities among them. One of the most prevalent pesticides across the board is Boscalid, a fungicide that consistently appears. Other substances like Azoxystrobin and Epoxiconazole are also widely used across many countries, suggesting a common reliance on certain fungicides.

**Figure 10.**
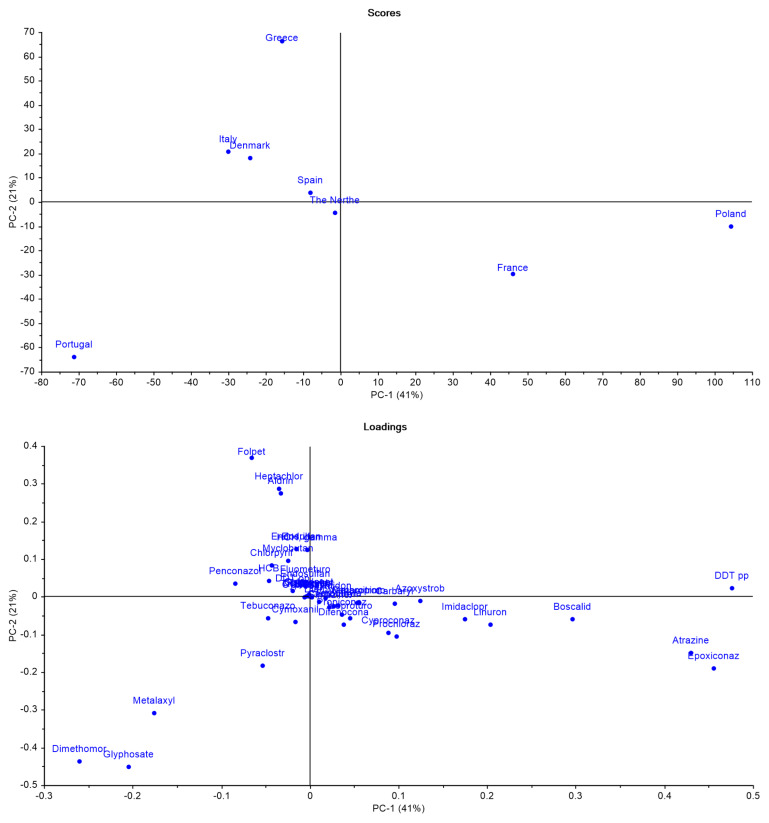
Principal Component Analysis (PCA) scores and loadings of pesticide use across selected European countries.

To further explore whether there were identifiable patterns among countries, PCAs were performed. The analysis considered only pesticides that were studied across all selected countries, ensuring a consistent basis for comparison ( Figure 10).

The PCA ( Figure 10) results explain 62% of the variance information from data and reveal differences between countries. Poland and France stood out along the PC-1 axis, suggesting distinct pesticide profiles compared to other countries. Portugal also showed a unique pattern, positioned far from the rest, indicating a particular use of pesticides. In contrast, Italy, Denmark, Spain, and the Netherlands were more clustered together, suggesting some similarities in their pesticide use.

When looking at the loadings ( Figure 10), certain pesticides were strongly associated with these country-specific patterns. Epoxiconazole and p,p’-DDT were linked to Poland and France. Folpet, Aldrin, and Heptachlor contributed to the differentiation of Greece. Meanwhile, Glyphosate and Dimethomorph appeared more closely tied to Portugal, reflecting its isolated position in the plot.

Despite some clustering among countries, the PCA results ( Figure 10) highlight the absence of a clear geographical pattern in pesticide use. While certain regional similarities exist, the differences between others, such as Portugal and Poland, emphasize the complex, non-linear nature of pesticide distribution across Europe. The 2018 LUCAS also concluded that aggregation by country reveals differences across EU nations, though no clear geographical pattern emerges between macro-regions ^46^.

Although differences in the use of approved pesticides were expected, what is more surprising is the presence of non-approved ones. Hexachlorobenzene (HCB) and Chlorpyrifos were among the banned compounds most frequently detected in several countries ( Figure 8). Chlorpyrifos was banned in 2020, coinciding with the period when the analysed samples were collected ^75^. This likely explains its recurring presence in the top 10 lists across different regions. In contrast, HCB has a much longer and more complex history. Introduced as an agricultural pesticide in 1945, its use was banned in the EU in 1981 ^76^. However, due to its high persistence with a typical DT50 of roughly 2000 days, HCB continues to be a prevalent contaminant ^69^. Additionally, it is still used as an industrial chemical and is released into the environment through waste incineration, contributing to its ongoing environmental presence ^76^.

**Figure 11.**
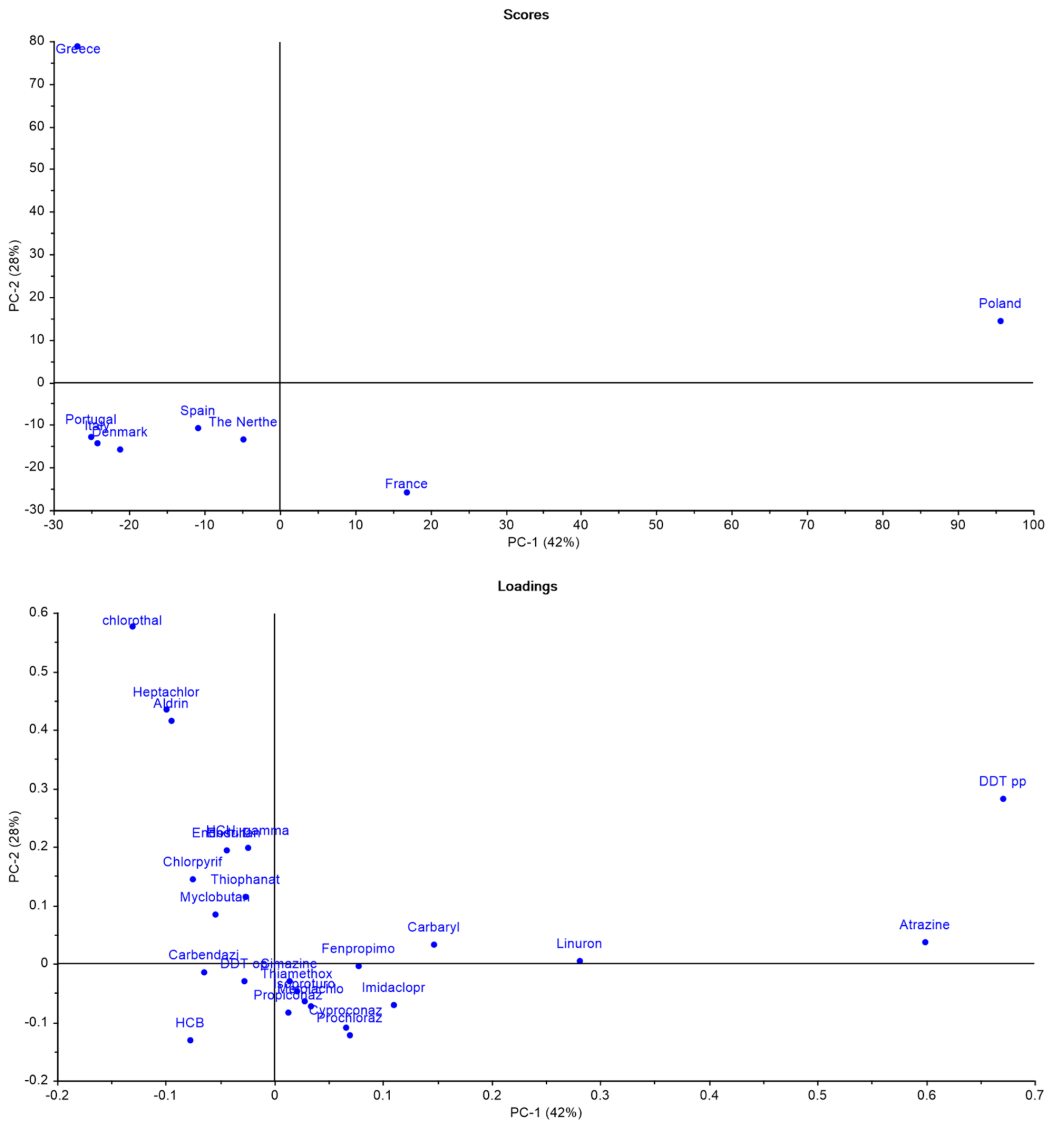
Principal Component Analysis (PCA) scores and loadings of non-approved pesticide use across selected European countries.

Using the same methodology as before, a second PCA was conducted, focusing only on non-approved pesticides ( Figure 11). The results explain 70% of the variance information from data and highlight several key findings. Greece stands out due to its strong association with banned substances such as Aldrin, Heptachlor, and Chlorothalonil. Poland also showed a significant link to non-approved pesticides, particularly aligning with p,p’-DDT and Atrazine. Interestingly, Portugal, despite its previously isolated position in the PCA ( Figure 10), appeared to be less connected to these banned substances.

Chlorothalonil, like Imidacloprid, Prochloraz, and Chlorpyrifos, was banned during the period when the sampled soils were collected, in this case, in 2019, likely explaining its presence ^18^. However, some European countries still allow its use today ^69^. The situation with Aldrin and Heptachlor is more complex. Both substances were banned in the European Union long before the soil samples were collected. Aldrin was banned in 1991 and Heptachlor in 1984 ^77, 78^. This raises the question on how are they still being detected.

A 2021 study in Albania, a country that shares border with Greece (country where these compounds were most prevalent), found traces of Aldrin and Heptachlor in agricultural soils from twelve different locations in Belsh ^79^. This is puzzling, considering that both substances were banned under the 2001 Stockholm Convention on Persistent Organic Pollutants ^79^. Their presence raises important questions: Are these compounds a legacy of past contamination, or is there an ongoing source?

As for the findings in Poland, the detection of Atrazine, a pesticide banned in 2003, was particularly unexpected ^80^. Atrazine is classified as non-persistent, with a DT50 (field) of just 26 days, meaning it typically degrades quickly under normal conditions ^81^. Its presence suggests potential sources beyond residual contamination from past use, such as cross-border contamination or long-range transport mechanisms. Notably, in 2018 (year included in the period when the sampled soils were collected), the EU exported 1,425 tonnes of Atrazine, including shipments to Ukraine, Poland’s neighbouring country ^81^. Could this be the explanation for the contamination?

### Metabolites across Europe

Beyond the active substances themselves, the breakdown products, or metabolites, of these also play a crucial role. Although most research has been focused on parent compounds, there is increasing recognition that metabolites can persist in the environment and exhibit their own toxicity. Despite this, studies addressing the environmental fate and behaviour of pesticide metabolites are less prevalent. A total of 90 metabolites were analysed in this study, significantly fewer than the 455 parent compounds, reflecting this research gap ^17, 46, 53– 68^.

Most metabolites were studied in only a single article, limiting the ability to fully assess their distribution across Europe. However, targeted studies provide valuable insights into the relationship between certain parent compounds and their transformation products ^17, 46, 53– 68^.

For example, a study analysing 40 sampled soils sheds light on Atrazine and its metabolites ^62^. Atrazine-desethyl was detected with a mean concentration of 0.2 µg/g and a maximum of 0.503 µg/g, while Atrazine-deisopropyl exhibited a mean concentration of 0.213 µg/g and a maximum of 0.321 µg/g. By comparison, the parent compound Atrazine had a lower mean concentration of 0.193 µg/g but reached a much higher maximum concentration of 1.75 µg/g.

In contrast, findings from the 2018 LUCAS, which analysed over 3000 soils, reveal a broader pattern ^46^. The maximum concentrations of herbicide metabolites were consistently higher than those of their parent compounds, reinforcing the need for large-scale studies to provide reliable statistical conclusions. Notably, even in smaller studies, metabolites frequently showed higher mean concentrations, underscoring their persistence and potential for long-term soil accumulation ^62^.

**Figure 12.**
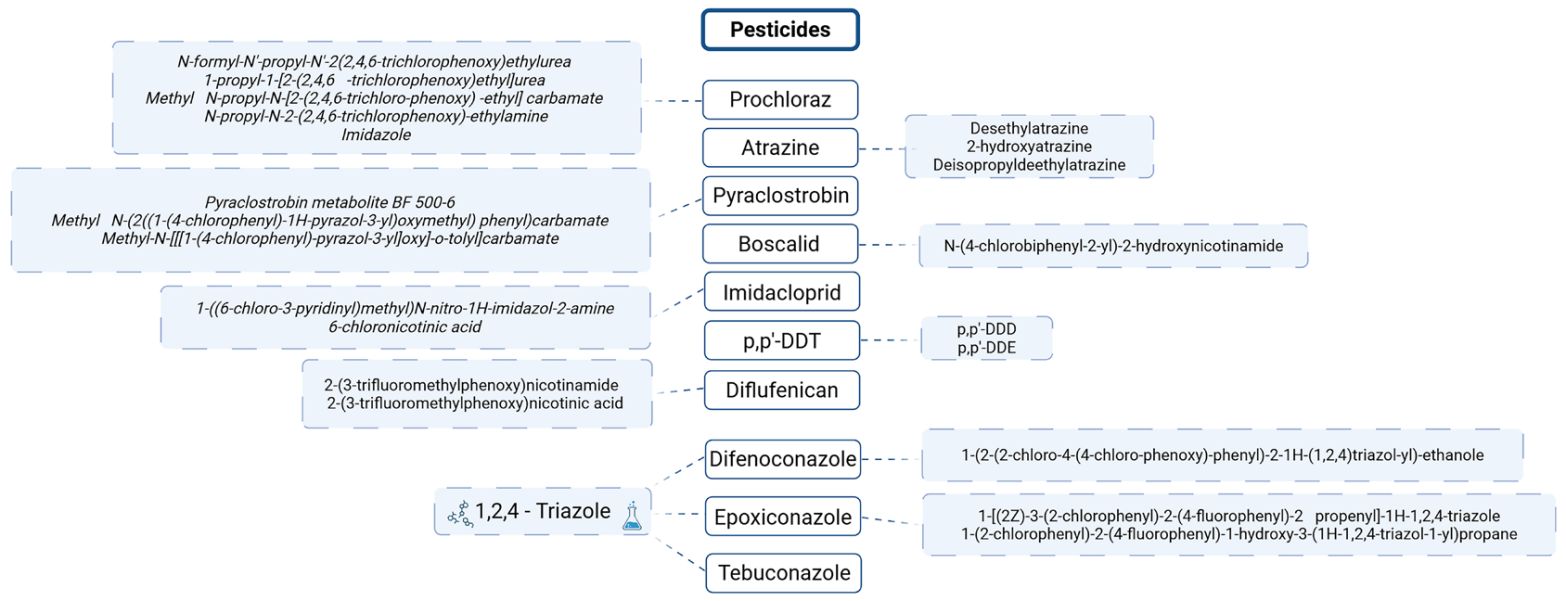
Metabolites of the TOP 10 pesticides of concern across Europe. Created in BioRender. Carvalho, R. (2025) https://BioRender.com/jsrt3v6.

Using the previously discussed top 10 pesticides of concern across Europe ( Figure 7) as a reference, Figure 12 highlights their associated metabolites ^69^.

**Figure 13.**
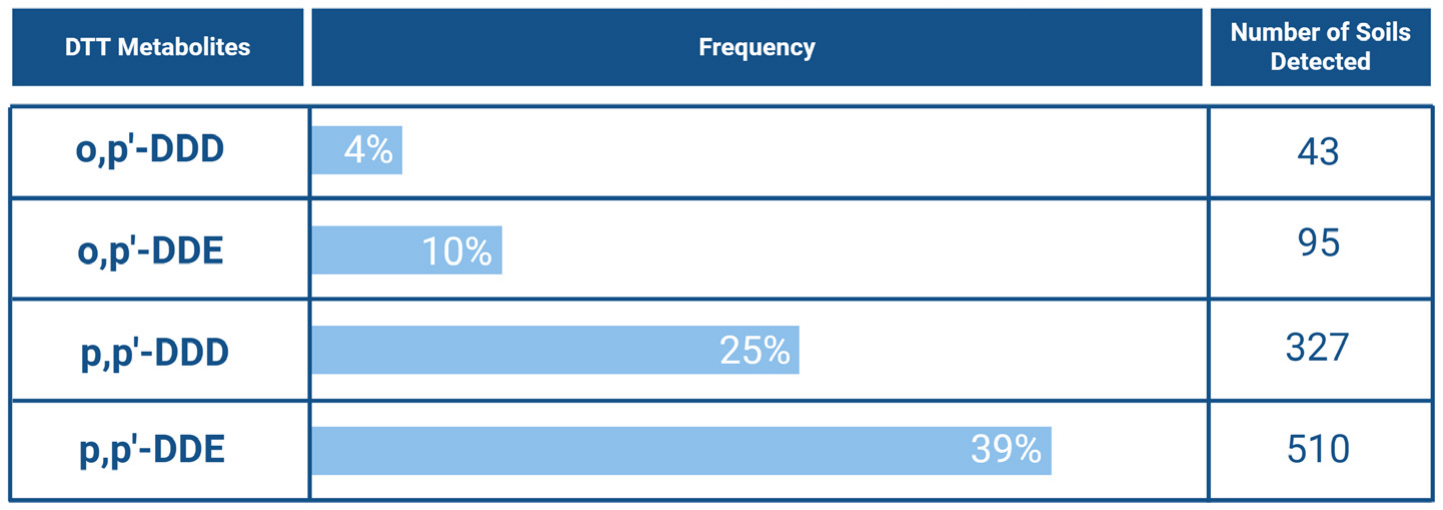
DDT metabolites, and their respective incidence across European soils ^10, 42– 58^. Created in BioRender. Carvalho, R. (2025) https://BioRender.com/e0ffgrr.

Building on this discussion of pesticide metabolites, a key example is DDT, a pesticide whose legacy illustrates the persistent nature of both parent compounds and their transformation products in the environment. DDT was once widely used to fight diseases like malaria and to control insect infestations ^82^. Although banned in 1972 in EU Member States, DDT and its breakdown products, DDD (dichlorodiphenyldichloroethane) and DDE (dichlorodiphenyldichloroethylene), continue to persist in soils across Europe ^17, 46, 53, 55, 61, 63– 66, 68^. There are two isomeric forms of DDT - p,p’-DDT and o,p’-DDT. Of the two, p,p’-DDT was the most frequently detected, being found in 408 sampled soils (31% incidence), while o,p’-DDT appeared in 72 samples (7%). Similarly, its metabolites, p,p’-DDE and p,p’-DDD, are also frequently detected, with p,p’-DDE found in 510 sampled soils (39%) and p,p’-DDD in 327 sampled soils (25%) ( Figure 13). These numbers demonstrate how DDT affects the environment, even decades after its ban. Notably, the 2018 LUCAS identified p,p’-DDE as one of the most recurrent compounds in European soils and a significant contributor to toxicity ^46^.

The persistence of pesticide metabolites, as illustrated by DDT, is not an isolated phenomenon. Many other pesticides also break down into multiple transformation products, some of which are shared across different compounds. For example, Cyproconazole has at least seven known metabolites in soil, including triazole lactic acid, triazole pyruvic acid, and 1,2,4-triazole. Similarly, Epoxiconazole degrades into various compounds, such as 1,2,4-triazole and other triazole-related metabolites. Tebuconazole and Difenoconazole also break down into 1,2,4-triazole, making it a common metabolite across several fungicides. This highlights the recurring presence of 1,2,4-triazole in the degradation pathways of many fungicides ^69^.

***The case of 1,2,4 triazole***

As a result of their effectiveness in treating fungal infections, triazole fungicides have become some of the most widely used and profitable fungicides globally. Their mode of action involves inhibiting sterol 14-α-demethylase (CYP51), a crucial enzyme in the synthesis of ergosterol, which is necessary for maintaining fungal cell membranes and the development of fungal cell walls ^83^.

**Figure 14.**
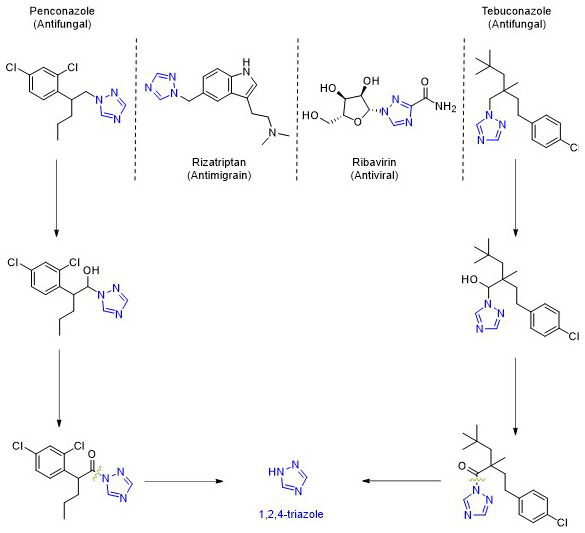
Compounds with 1,2,4-triazole in the skeleton and respective properties. Proposed degradation pathways for Penconazole and Tebuconazole.

These fungicides, as well as many pharmaceutical drugs ^84^, share a common structural feature: the 1,2,4-triazole ring. This means that when both pesticides and pharmaceuticals degrade, they release 1,2,4-triazole into the environment ( Figure 14). Depending on the compound, between 3% and 44% of these substances break down into this metabolite ^85^.

1,2,4-Triazole presents environmental risks, such as reproductive toxicity and neurotoxicity, with additional concerns about its potential to leach into groundwater ^85, 86^. Although most triazole fungicides are hydrophobic and less likely to leach, 1,2,4-triazole, due to its high-water solubility (>700 g/L), poses a greater risk of leaching ^86, 87^.

**Figure 15.**
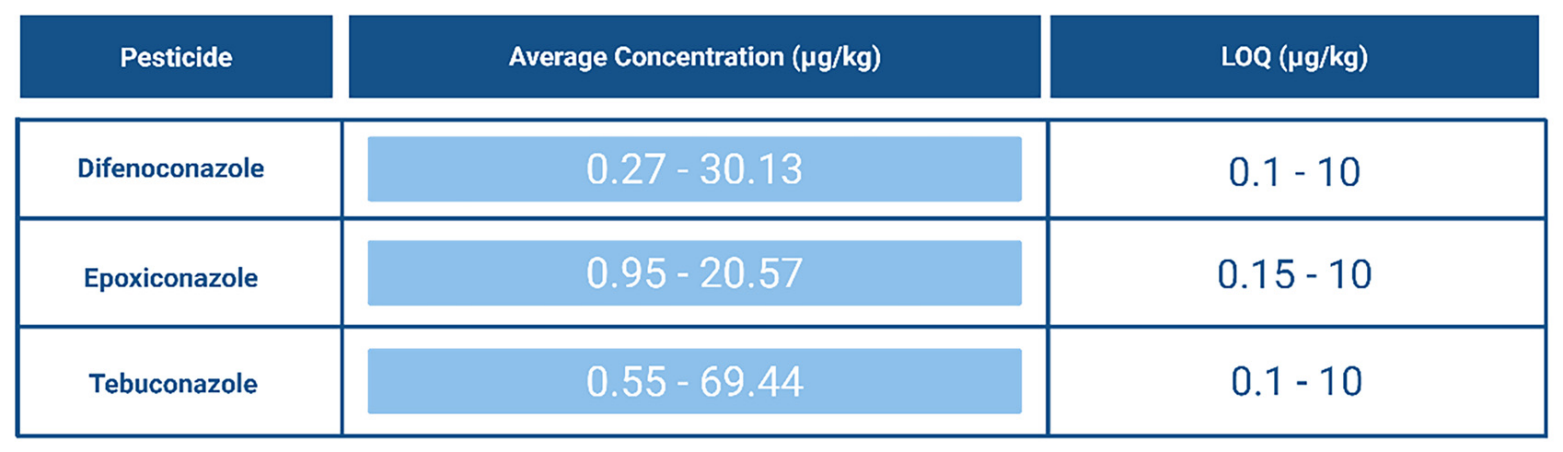
Average concentration (μg/kg) and Limit of Quantification (LOQ) (μg/kg) of pesticides most detected in Europe that degrade into 1,2,4-triazole ^10, 47, 53, 54, 59^. Created in BioRender. Carvalho, R. (2025) https://BioRender.com/f8tvrzb.

The accumulation of 1,2,4-triazole in soils can lead to significant negative effects, particularly because many different compounds break down into this common metabolite. This buildup is concerning and requires close monitoring, especially since several pesticides from the Top 10 pesticides list degrade into 1,2,4-triazole ( Figure 12). Figure 15 provides an overview of the concentrations at which these pesticides were detected.

In studies, 1,2,4-triazole has been analysed using Liquid Chromatography Tandem Mass Spectrometry (LC–MS/MS,) though challenges have been reported due to its low molecular weight (69.1 g/mol) and mass spectrometric interferences from co-eluting matrix compounds. Researchers have successfully used multiple reaction monitoring (MRM) transitions (70/70, 70/43) to detect 1,2,4-triazole. In one study, a limit of detection (LOD) of 0.5 μg/kg was reported, along with a limit of quantification (LOQ) of 1.1 μg/kg. For propiconazole (a parent compound), the LOD was 2.4 μg/kg and the LOQ 4 μg/kg ^85^. As shown in Figure 15, LOQ values can vary between methods for the same compound. Nevertheless, literature shows that 1,2,4-triazole can be successfully analysed with relatively low LOQ values. Given its associated challenges and potential for accumulation, it is always wise to monitor this compound.

To enhance monitoring and potentially reduce analysis costs, 1,2,4-triazole could serve as a practical indicator of pesticide contamination in soils. Notably, the 1,2,4-triazole fragment is unknown in any natural product, making it a unique and reliable marker for contamination ^88^. Since numerous pesticides and other contaminants degrade into 1,2,4-triazole, and considering the robust limits of detection and quantification achievable for this compound, its monitoring offers a feasible approach for initial screening ^85^.

A threshold concentration for 1,2,4-triazole could be established to guide subsequent actions. When levels exceed this threshold, more detailed analyses, either targeted to specific pesticides or employing broader non-targeted approaches, could be recommended to identify the precise contaminants present. This indicator-based strategy would streamline soil testing by focusing resources on a single representative metabolite. Comprehensive analyses would be initiated only when contamination is indicated, thereby optimizing the overall cost and efficiency of soil assessment processes.

***Glyphosate and AMPA***

Given the impact and potential use of metabolites, like 1,2,4 triazole, it is essential to also consider others, like AMPA (Aminomethylphosphonic acid).

Although Glyphosate did not make the top 10 list of most concerning pesticides, it is still noteworthy. According to the World Health Organization classification, Glyphosate is considered slightly hazardous (Class III). Additionally, it is non-persistent in the environment and has low bioaccumulation potential. These factors have led to the prioritization of other pesticides, such as Difenoconazole which, despite being detected in fewer soils and with a lower incidence, has a higher bioaccumulation potential, is classified as moderately hazardous (Class II), and is moderately persistent ^69^.

However, Glyphosate still demands attention, as it was detected in 161 sampled soils (18% incidence), while its metabolite AMPA was found in 396 soils (44% incidence). This highlights a key concern: although Glyphosate is non-persistent and breaks down relatively quickly, it degrades into AMPA, a more toxic compound ^32^.

In studies that analysed both compounds, AMPA consistently appeared at concentrations similar to or exceeding those of Glyphosate ^17, 53, 60, 61^. For example, in two of the studies, AMPA was detected at roughly double the median concentrations of Glyphosate, with values such as 0.11 µg/g versus 0.06 µg/g and 0.06 µg/g versus 0.02 µg/g ^60, 61^. This pattern is further exacerbated by the fact that AMPA is detected much more frequently than Glyphosate.

Although these concentrations remain below acute toxicity thresholds (65 µg/L for AMPA and 6500 µg/L for Glyphosate) ( Figure 2), AMPA’s significantly higher toxicity and frequent presence in larger quantities are cause for concern ^32^. Its persistence and potential for accumulation in soils, if left unmonitored, could pose increasing risks to soil ecosystems over time.

An additional issue is the development of herbicide resistance, to which Glyphosate widespread use contributes significantly, but it is not the only pesticide responsible ^89^. The 2018 LUCAS further underscores the widespread presence of Glyphosate and its metabolite AMPA in European soils ^46^. AMPA was frequently detected, often as the sole residue found in some locations. This reinforces the observation that AMPA is more commonly detected than Glyphosate, highlighting its persistence in the environment. While the survey concluded that AMPA is not significantly associated with high-risk sites or major contributors to risk indicators, its prevalence raises concerns about its potential for accumulation over time. Coupled with its higher toxicity compared to Glyphosate, these findings emphasise the need for continued monitoring and evaluation of AMPA’s long-term environmental impact, even if immediate risks appear low.

### What can be improved?

To better understand and to improve the management of pesticide contamination in soils, key areas require attention and enhancement.

The effectiveness of current pesticide detection methods must be assessed. Many pesticide analyses rely on tandem mass spectrometry (MS/MS), which, despite its efficacy, is prone to issues such as co-elution and shared ion interference. As a result, some pesticides may be easier to detect, while others may go almost undetected, which could lead to false negatives.

It is challenging, if not impossible, to rely on a single sample preparation method and one analytical technique for all the pesticides due to their variability. Because each pesticide has unique chemical properties, context-specific methods are necessary to guarantee accurate detection. This is reflected in the literature, where studies employ various preparation methods, each with its detection limits and challenges. For instance, even within a single study, 3 different methodologies were applied based on the specific characteristics of the pesticide residues being analysed ^17^.

Because different studies employ different preparation and analytical techniques, the lack of standardisation across them makes it difficult to compare results. Although creating a universal method for all pesticides is unrealistic due to their diverse nature, it would be beneficial to have a single standardised preparation method for each pesticide. The same applies to analytical techniques, where variations in equipment and sensitivity further hinder uniformity. Nonetheless, establishing a consistent approach per pesticide could significantly improve the reliability and comparability of data across different research efforts.

Another critical issue is the normalisation of pesticide incidence data. Many studies do not investigate the same range of pesticides, and some rely on smaller sample sizes for certain compounds. For example, if only 159 sampled soils are analysed for a pesticide, as was the case for Ipridione, and it is detected in 28, the incidence rate appears high (18%), but this may not accurately represent its broader distribution.

To address these challenges, a standardised, large-scale investigation should be established. The LUCAS 2018 highlighted that knowledge suggesting expanding his assessment from 3,473 sampled soils in the EU to the total extent of LUCAS points for 2022 (~40,000) ^46^. This expansion would not only increase knowledge about the number of contaminants present but also promote better interaction with countries to understand key management practices and stay aware of recent contamination patterns.

While it may not be feasible to analyse every pesticide in use, being able to access detailed records where farmers report the specific pesticides used, their application timing, and other relevant data could significantly improve the accuracy of contamination assessments. Including pesticide metabolites in these investigations is essential, as they can be more toxic or persistent than their parent compounds.

Regular monitoring would also be beneficial in tracking trends over time, assessing degradation pathways, and identifying contamination patterns. This information would help direct remediation efforts to areas of highest concern.

Legislation must also evolve to address the issue of inert compounds in pesticide formulations. These compounds, though not intended to have a direct effect on pests, can alter the behaviour of active ingredients and may pose health and environmental risks. Current regulatory frameworks often overlook the impact of inert ingredients, leading to underestimation of their potential dangers. Equally important is the fact that pesticides are rarely used in isolation; mixtures of multiple active substances are far more common in real-world applications. This complexity should not be ignored, since we have not yet achieved comprehensive knowledge about the cumulative or synergistic effects that these mixtures may have on human health and other living organisms ^17, 53, 90^.

### Pesticides know no borders

The European Union’s pesticide regulations are designed to protect both environmental and human health. However, the persistence and movement of these substances reveal a significant challenge: pesticides do not recognize political borders. This problem is emphasized by the existence of banned pesticides in areas where their use has long been prohibited.

***The challenge of cross-border contamination and policy gaps***

The fact that pesticide regulations are applied at the national or regional level, whereas environmental processes function on a much larger scale, is one of the main issues with them. Through a variety of processes, such as soil transport, water runoff, and atmospheric drift, pesticides can spread well beyond the areas where they are intended to be applied. One study that examined the presence of 209 pesticides in soil, crops, surface water, surface air, sediment, and even indoor dust samples provided evidence of this 90. Even if a country bans a specific substance, neighbouring regions where its use remains permitted can become indirect sources of contamination.

This creates a regulatory gap: even if a country has strict ban on the use of a particular pesticide, it may still be exposed due to factors beyond its control. Additionally, there is another critical question, given that our understanding and knowledge of pesticide degradation under field conditions is still limited: are experimentally measured DT50 values truly representative of the "environmental reality"? This issue highlights the need for post-approval monitoring programs in ‘real-life’ scenarios.

There are still some policy gaps in spite of EU’s efforts to regulate pesticides. Some pesticides are still manufactured for export, even though they are banned within the EU. Additionally, stockpiles of previously approved pesticides present a dilemma. When a pesticide is banned, what happens to the remaining supplies? Can they still be used until exhausted? These unresolved questions expose gaps in current policies that allow continued exposure, even when official approvals have been revoked.

The possibility of illicit pesticide use is another challenge. Banned substances may still find their way into agricultural practices, for a variety of reasons, such as the availability of old stock or limited access to alternatives. In some cases, the use of non-approved pesticides may be consequence of economic pressures or misinformation, rather than deliberate use.

We require more knowledge to deal with all of these problems. Better education and support are also required to give farmers who are faced with difficult decisions, alternatives.

## Conclusion

The widespread contamination of European soils by both approved and banned pesticides highlights a persistent environmental challenge that extends beyond national regulations.

Because of the complexity of environmental transport mechanisms and the legacy of past applications, harmful substances continue to be detected, despite strict policies. Notably, pesticides do not recognize political borders, as banned substances still appear in areas where their use has long been prohibited. Most likely this is a result of soil persistence, cross-border movement, and occasionally illicit applications.

Furthermore, current regulatory frameworks tend to focus on the active ingredients in pesticides, often ignoring the effects of their metabolites and inert co-formulants, which can be just as toxic, if not more so. The necessity for more comprehensive risk assessments taking into account the full spectrum of contaminants associated with pesticides is highlighted by the high detection rates of AMPA and other breakdown products.

Moving forward, it will be crucial to improve detection techniques using markers like 1,2,4-triazole and expand soil monitoring initiatives, as well as enforce stricter regulations over pesticide trade and usage. Mitigating soil degradation requires a shift to sustainable agricultural practices and enhanced international cooperation on pesticide regulation. The persistence of these pollutants will continue to endanger human health and the environment for future generations unless immediate action is taken.

## Ethics and consent

“Ethical approval and consent were not required”.

## Data Availability

All supplementary materials used in the review are publicly available via Knowledge Network for Biocomplexity ^91^. The dataset includes: (1) Supplementary_Material.docx: Structured summary tables of the top 10 detected pesticides by country, including regulatory status, type, detection frequency, and number of soils in which each was found; (2) Supplementary_Material.xlsx: The raw and organized spreadsheet used to extract and structure pesticide detection frequency data from included studies; (3) PRISMA_2020_Checklist.docx: Completed PRISMA 2020 checklist documenting adherence to systematic review standards, including study selection, data extraction, synthesis methods, and risk of bias considerations. The repository can be accessed at: doi: 10.5063/F1Z899WP. This work is dedicated to the public domain under the Creative Commons Universal 1.0 Public Domain Dedication.
