# Supplementary material for: Soil contamination in Europe unveiled: A review of pesticides and metabolites to watch
Source: Open Res Eur. 2025 Nov 24;5:257. Originally published 2025 Aug 27. [Version 2] doi: 10.12688/openreseurope.20475.2 (PMC12648036; doi:10.12688/openreseurope.20475.2)
Supplement: Peer review history

# Reviewer report: Reviewer response for version 2

**DOI:** 10.21956/openreseurope.23736.r64756

**Published:** 2025-11-25

**Authors:** Zijian Li

**Affiliations:** 1 Sun Yat-Sen University, Shenzhen, China

The quality of the paper has been substantially improved.

Are the rationale for, and objectives of, the Systematic Review clearly stated?

Yes

Is the statistical analysis and its interpretation appropriate?

Yes

Are sufficient details of the methods and analysis provided to allow replication by others?

Yes

Are the conclusions drawn adequately supported by the results presented in the review?

Yes

Reviewer Expertise:

Pesticides

I confirm that I have read this submission and believe that I have an appropriate level of expertise to confirm that it is of an acceptable scientific standard.

# Reviewer report: Reviewer response for version 1

**DOI:** 10.21956/openreseurope.22156.r59829

**Published:** 2025-09-25

**Authors:** Mohamed Hamza EL-Saeid

**Affiliations:** 1 King Saud University, Riyadh, Saudi Arabia

This review clarifies that the pesticide contamination trends across Europe were assessed by analysing published data from 5193 sampled soils collected between 2015 and 2022. By raking pesticides based on detection frequency, persistence and toxicity, key concerns were brought to attention, including the presence of banned substances, such as p,p’-DDT (detected in 31% of sampled soils) and Atrazine (17%), as well as high detection rates of currently approved pesticides like Boscalid (36%) and Epoxiconazole (32%).

Are the rationale for, and objectives of, the Systematic Review clearly stated?

Yes

Is the statistical analysis and its interpretation appropriate?

Not applicable

Are sufficient details of the methods and analysis provided to allow replication by others?

Yes

Are the conclusions drawn adequately supported by the results presented in the review?

Yes

Reviewer Expertise:

Environmental Pollution

I confirm that I have read this submission and believe that I have an appropriate level of expertise to confirm that it is of an acceptable scientific standard.

# Reviewer report: Reviewer response for version 1

**DOI:** 10.21956/openreseurope.22156.r59823

**Published:** 2025-09-23

**Authors:** Zijian Li

**Affiliations:** 1 Sun Yat-Sen University, Shenzhen, China

The paper pulls together a large body of evidence on pesticide contamination in European soils, covering both active substances and metabolites. The scope is broad, the methodology is clear, and the findings are presented in a way that is both accessible and relevant to ongoing policy discussions. I found the paper to be well-written and thoughtfully structured, and I believe it will be of real interest to researchers.

 Suggestions:

 Figures 6 and 7: The main trends come across, but the figure could be polished.

 It would be useful to briefly note in the methods how PCA data were standardized.

 The policy section could be strengthened with one or two specific recommendations—for instance, highlighting 1,2,4-triazole as a practical screening marker for soil monitoring.

 A short comparison with similar global reviews could help to place the European findings in context.

 Figure 11: In the current version, some of the country names overlap with each other, making them hard to read.

Are the rationale for, and objectives of, the Systematic Review clearly stated?

Yes

Is the statistical analysis and its interpretation appropriate?

Yes

Are sufficient details of the methods and analysis provided to allow replication by others?

Yes

Are the conclusions drawn adequately supported by the results presented in the review?

Yes

Reviewer Expertise:

Pesticides

I confirm that I have read this submission and believe that I have an appropriate level of expertise to confirm that it is of an acceptable scientific standard, however I have significant reservations, as outlined above.
